# Transition shocks during adulthood and health a few decades later in post-socialist Central and Eastern Europe

**DOI:** 10.1186/s12889-020-08839-7

**Published:** 2020-05-15

**Authors:** Anikó Bíró, Réka Branyiczki

**Affiliations:** 1 Health and Population “Lendület” Research Group at the Centre for Economic and Regional Studies, Tóth Kálmán utca 4, Budapest, 1097 Hungary; 2 Central European University and TÁRKI, Budapest, Hungary

**Keywords:** Aging, Health status, Post-socialist transition

## Abstract

**Background:**

Health of the population of post-socialist Central and Eastern European (CEE) countries lags behind the European Union average. Our aim in this paper is to analyse the link between transition shocks and health two-three decades later.

**Methods:**

We use retrospective data from the Survey of Health, Ageing and Retirement in Europe. We estimate the implications of stressful periods, financial hardships and job loss occurring around the transition (1987–1993) on subjective and objective measures of health in 2017. We compare these implications across groups of CEE countries and with the health implications of similar difficulties reported by individuals from Western Europe. We also compare the health implications of difficulties occurring around the transition to difficulties occurring before or after the transition.

**Results:**

In the CEE region there is a peak in the timing of difficulties around the transition. Stressful periods, financial difficulties and job loss around the period of transition are generally associated with worse subjective and objective health at older ages in all groups of CEE countries, even after netting out the effect of childhood health and demographic factors. However, the consequences of hardships due to the transition are not specific, health implications of these difficulties seem to be similar to the implications of other shocks possibly unrelated to the transition.

**Conclusions:**

The high fraction of individuals experiencing stress, financial difficulties and job loss around the transition contributed to the current health disadvantage in the CEE region. As similar shocks in the West and before or after the transition had similar health implications, our results draw the attention to the long-lasting impacts of psychosocial stress and financial hardship during adulthood on later health over the life course.

## Background

Health of the population of post-socialist Central and Eastern European (CEE) countries lags behind the European Union average [1]. Previous results from the literature [2, 3] suggest that the East-West health gap can partly be explained by differences in health behaviours and psychosocial factors. Health behaviours and psychosocial factors are likely to be related to living conditions during and after the communist era, and to the transition itself. Our aim in this paper is to analyse the link between transition shocks and health 2–3 decades later. Our main research question is whether experienced psychosocial stress as an adult around the transition period had adverse health implications observable at older ages in the life course.

After the fall of the Berlin wall, the dictatorship of the Communist Party came to an end in CEE around 1990, and the Soviet Union dissolved in 1991. The transition implied a dramatic restructuring of the economy and of the social security system. Privatisations took place, prices were no longer fixed, and job security disappeared [4]. Overall, the stress and financial hardship due to job losses, increasing insecurity and rising inequalities affected the health status of the population in CEE [5–7].

A severe transformational recession was a common phenomenon in the region [8–10], however, the pace of transformation varied from gradual transition to shock therapy across countries [11–13]. Bohle and Greskovits (2012) grouped post-socialist capitalist systems into neoliberal (Baltic countries) and embedded neoliberal (Visegrad states) types. The former group combined radical marketization with minimal social protection, while the latter compensated the losers of marketization by more generous welfare states [14].

So far, studies investigating the East-West health gap and the implications of the transition focused mainly on mortality. Zatonski (2007) documents that adult mortality rates in post-socialist CEE countries started to diverge from Western countries in the 1960s [15]. Also, during the period of transition, adult mortality rose particularly rapidly in the post-socialist CEE countries [6], especially in the Baltics. Rapid and mass privatization was found to be a significant factor in the declines in life expectancy and in the increase in alcohol-related deaths, heart disease, and suicide rates, also pointing to the role of excess psychosocial stress [5, 7, 16].

Less scholarly attention has been paid to the long-term ramifications over the life course, in particular to the link between stressors of the post-socialist transition and later health outcomes at the individual level. Taking a life course perspective has gained prominence in several fields of social science from sociology [17, 18] to gerontology [19, 20] with the common emphasis on assessing the impact of changes over a long period of lifetime, assuming that early events and impulses affect later life outcomes [18]. Similarly, the life course theory of health focuses on how health inequalities evolve due to socioeconomic circumstances and hardships [21–23].

Our paper relates to studies of health trajectories following psychosocial stress and economic strain in adulthood [24, 25]. We identify the transition from one-party rule and socialism to democracy and market-economy as an immense source of psychosocial stress due to rapidly increasing job insecurity, inequalities and general uncertainty that may have adverse health implications that unfold over the life course.

Overall, our contribution to the literature is threefold: first, we are among the few [26] who study health implications of the transition at the micro level, linking experienced stressors of the transition with both self-rated and more objective health outcomes at the individual level. Second, we take a life-course perspective and assess long-term health implications. Finally, we make use of a harmonised dataset across European countries, which enables us to compare post-socialist countries and to contrast the shocks of the transition to stress and economic strain at other times and elsewhere, unrelated to a system change. Since during the transition both the political and the economic system changed drastically, and not only the economic output or employment dropped within a stable socioeconomic system, it is not obvious whether shocks related to the transition or to an economic downturn are comparable in terms of their health implications.

We exploit individual level life history data to increase our understanding on how difficulties, such as stress, financial hardship and job loss around the transition relates to later population health. We demonstrate the present East-West gap in health outcomes of the population above age 50, and test associations between difficulties around the system change and later health of the individual. We analyse differences in these associations across three groups of CEE countries with different pace of transformation and varying post-socialist market economies: the Visegrad countries, Baltic countries, and Southern CEE countries. This division of the CEE countries is in line with the typology of Bohle and Greskovits (2012) and also with the regions defined by Dingsdale (2000) [27], except for Slovenia and the Visegrad countries that belong to the same group. Since despite the similarities, there are non-negligible economic, political, social and cultural differences among the CEE countries within the three country groups, we also look at the associations between individuals’ health and transition-related difficulties on the country level.

## Methods

### Data source and country coverage

We analyse the third and seventh waves of the Survey of Health, Ageing and Retirement in Europe (SHARE)^1^ [28], ,which is a cross-national panel database of micro data on health and socio-economic status of individuals aged 50 or older covering 27 European countries and Israel [29, 30] . The third and seventh waves include retrospective questions about respondents’ life history, such as employment history, periods of stress and financial difficulties, and health at younger ages. Data were collected in 2009 and 2017 respectively, thus even the youngest cohorts of the sample were already of active age during the times of transition. The seventh wave of SHARE questionnaire contains a retrospective questionnaire for all respondents who did not participate in the third wave, as well as a regular panel questionnaire for all respondents who already answered the retrospective questions in the third wave. Thus, each SHARE respondent who participated in the seventh wave answered the retrospective questions exactly once (either in the third or the seventh wave). We group the countries into post-socialist CEE countries (Bulgaria, Croatia, Czech Republic, Estonia, Hungary, Latvia, Lithuania, Poland, Romania, Slovak Republic, Slovenia) and the rest, labelled as ‘West’ (Austria, Belgium, Cyprus, Denmark, Finland, France, Germany, Greece, Ireland, Israel, Italy, Luxembourg, Malta, Netherlands, Portugal, Spain, Sweden, Switzerland). We split the German sample according to the place of residence on 1st November 1989 (i.e. before the Berlin wall came down).

We split the post-socialist CEE countries (except for East Germany) further into three groups: the Visegrad countries (V4: Czech Republic, Hungary, Poland, Slovak Republic), Baltic countries (Estonia, Latvia, Lithuania) and Southern countries (Bulgaria, Croatia, Romania, Slovenia).

### Measures

We assess the current health (as measured in 2017, the 7th wave of the SHARE data) of the 50+ population with several indicators. Self-rated general health is measured on a 5-point Likert scale from excellent to poor, which is a strong predictor of morbidity and mortality [31]. From this variable, following the standard approach in the literature [32–35], we generate a binary indicator of poor health which equals 1 if the self-rated health is fair or poor, 0 otherwise. Other binary outcome variables indicate whether the respondent suffers from chronic or long-term health problems (long-term illness, henceforth), has a health problem that limits paid work, has certain conditions, such as heart problems, hypertension, diabetes, ulcer, cancer, and chronic lung disease (each condition is assessed by a separate dichotomous variable). Besides reported health conditions, dependent variables include obesity (Body Mass Index 30 or greater) and an indicator of grip strength, which was shown to explain old age disability [36]. Since grip strength, on average, varies by age, gender and the build of the individuals, we create a binary indicator of weak grip strength, which equals one if the grip strength is below the gender, 10-year age group and country specific median of grip strength. For the sake of brevity, we focus on the binary indicators of poor health and long-term illness in the main analysis, as two composite health indicators, with the indicator of long-term illness being more objective. We relegate the results on the other health measures to the Appendix.

To identify shocks around the transition, we look at retrospectively reported periods of stress and financial hardship that started between 1987 and 1993 and at reported end of jobs between 1987 and 1993 with at least 6 months of gap without employment or immediate retirement afterwards. The latter two indicators measure whether the respondent suffered from economic difficulties, while the stress variable may capture the general burden of uncertainties experienced during the system change as well. These binary measures of hardship are set to zero for those who do not report the analysed hardship ever (i.e. no stress, no hardship and no end of job with 6 months gap afterwards, except for retirement, respectively). Descriptive statistics are provided in Table 1.

**Table 1:** Descriptive statistics

	West		V4		South		Baltic	
	mean	std dev	mean	std dev	mean	std dev	mean	std dev
*Health in 2017*								
poor health	0.393	0.488	0.452	0.498	0.462	0.499	0.642	0.479
long-term illness	0.493	0.500	0.615	0.487	0.494	0.500	0.597	0.490
health limits work	0.213	0.410	0.324	0.468	0.182	0.386	0.318	0.466
any chronic disease	0.529	0.499	0.593	0.491	0.574	0.495	0.606	0.489
hypertension	0.393	0.488	0.480	0.500	0.472	0.499	0.474	0.499
heart problem	0.106	0.307	0.145	0.353	0.103	0.303	0.163	0.370
diabetes	0.130	0.336	0.161	0.368	0.117	0.321	0.100	0.300
ulcer	0.028	0.165	0.052	0.222	0.045	0.208	0.088	0.283
cancer	0.046	0.210	0.044	0.205	0.024	0.153	0.053	0.225
lung disease	0.058	0.234	0.050	0.217	0.045	0.207	0.056	0.231
obese	0.199	0.399	0.299	0.458	0.256	0.437	0.308	0.462
weak grip strength	0.531	0.499	0.517	0.500	0.540	0.498	0.534	0.499
*Hardship around transition*								
*(1 = yes; 0 = no hardship ever)*								
stressful period	0.137	0.344	0.081	0.272	0.104	0.305	0.123	0.328
financial difficulties	0.056	0.229	0.062	0.241	0.072	0.259	0.120	0.325
job ends with gap after	0.085	0.279	0.109	0.312	0.125	0.330	0.148	0.355
*Start of hardship conditional on hardship ever*								
*(1 = around transition; 0 = before/after transition)*								
stressful period	0.151	0.358	0.130	0.336	0.161	0.368	0.178	0.383
financial difficulties	0.129	0.336	0.158	0.365	0.155	0.362	0.271	0.445
job ends with gap after	0.242	0.428	0.355	0.479	0.451	0.498	0.314	0.464
*Individual characteristics*								
age in 2017	67.396	10.629	65.901	10.069	66.529	10.122	66.624	10.585
female	0.536	0.499	0.559	0.497	0.555	0.497	0.604	0.489
education (0 = primary, 2 = secondary, 3 = primary)	0.937	0.685	1.012	0.468	0.940	0.506	1.210	0.553
childhood health (1 = excellent to 5-poor)	2.192	1.036	2.200	0.981	1.970	0.964	2.616	1.044
hospitalisation during childhood	0.053	0.224	0.063	0.243	0.034	0.183	0.092	0.289
*Industry of last job prior 1987*								
agriculture, hunting, forestry, fishing	0.078	0.268	0.186	0.389	0.154	0.361	0.242	0.428
mining and quarrying	0.013	0.114	0.032	0.177	0.041	0.198	0.010	0.099
manufacturing	0.181	0.385	0.252	0.434	0.305	0.460	0.211	0.408
electricity, gas and water supply	0.019	0.137	0.022	0.146	0.023	0.150	0.024	0.153
construction	0.087	0.282	0.077	0.266	0.083	0.276	0.077	0.267
wholesale and retail trade	0.117	0.321	0.081	0.273	0.067	0.251	0.056	0.230
hotels and restaurants	0.033	0.180	0.021	0.143	0.020	0.141	0.022	0.147
transport, storage and communication	0.049	0.216	0.069	0.253	0.080	0.271	0.082	0.274
financial intermediation	0.031	0.172	0.010	0.097	0.006	0.079	0.008	0.091
real estate, renting and business activity	0.013	0.114	0.004	0.059	0.001	0.032	0.003	0.057
public administration and defence	0.093	0.290	0.051	0.220	0.033	0.178	0.033	0.180
education	0.081	0.272	0.071	0.257	0.050	0.217	0.102	0.303
health and social work	0.075	0.264	0.053	0.224	0.034	0.182	0.054	0.227
other community	0.130	0.336	0.072	0.259	0.102	0.303	0.075	0.263
*Total number of individuals*		43,424		12,310		10,025		8739

The number of observations vary across variables due to item non-response and due to sample restrictions for the hardship indicators. The indicator of the industry of last job is missing if no working period is reported

### Statistical models

We estimated multivariate logistic regressions of current health indicators, with binary measures of hardship during the transition as explanatory variables. We added the following confounding variables to the multivariate models that are likely to influence both health outcomes and our explanatory variable (shock indicator): age in 2017, gender, education (categorised as primary, secondary and tertiary, based on the international classification, ISCED-97), the industry code of the last job before the transition, and measures of childhood health (self-evaluated overall childhood health and a dummy for hospitalisation during childhood). To account for country specific differences, we included country dummies. We calculated cluster-robust standard errors, clustering on the country level, using the *vce (cluster clustvar)* option of Stata, as explained by Cameron and Trivedi (2009), section 3.3.5 [37]. All our results are based on weighted data, using calibrated individual weights. Hence, in the weighted sample, smaller countries have smaller weights. Also, with using the calibrated weights, we avoid bias due to unit nonresponse and panel attrition (see Malter and Börsch-Supan, 2015 [38] for details).

In the first group of models, we estimated the effects of the shocks around the transition on the subsample of CEE countries for each health outcome and type of hardship, allowing the effects to differ by subgroups of the CEE countries:

1 $$ \Pr \left({\mathrm{h}}_{\mathrm{igc}}=1\right)=\Lambda \left({\mathrm{s}}_{\mathrm{igc}}{\mathrm{D}}_{\mathrm{g}}{\boldsymbol{\upalpha}}_{\mathbf{1}}+{\mathrm{x}}_{\mathrm{igc}}{\boldsymbol{\upbeta}}_{\mathbf{1}}+{\gamma}_{\mathrm{c}}\right) $$

where Λ is the logistic function, *h*_*igc*_ is the binary indicator of current health problem of individual *i* living in country-group *g* and country *c*, *D*_*g*_ is a binary indicator of living in country group *g*, *s* is the indicator of hardship during transition, *x* is the set of confounding variables listed above, and γ_c_ captures the country effects. Our focus is on the exponential of the coefficient vector **α**_**1**_ (reported in Table 2), showing how the odds of a health problem in 2017 relates to having had hardships during transition in a specific country-group. Individuals who never had such hardships (according to the retrospective survey) serve as the comparison group.

**Table 2:** First and second groups of models - Health measures regressed on difficulties occurring between 1987 and 1993 in CEE country groups and in CEE and West

*First group of models*								
	Poor health	Long-term illness		Poor health	Long-term illness		Poor health	Long-term illness
Stress x V4	1.728***	2.610***	Fin. difficulties x V4	1.923***	2.112***	Job ends x V4	1.502***	1.419***
	[1.502–1.988]	[2.243–3.037]		[1.211–3.054]	[1.676–2.663]		[1.355–1.665]	[1.331–1.514]
Stress x South	2.042***	2.236***	Fin. difficulties x South	1.771***	1.549***	Job ends x South	1.599***	1.343***
	[1.607–2.596]	[2.052–2.438]		[1.263–2.484]	[1.111–2.159]		[1.430–1.788]	[1.222–1.476]
Stress x Baltic	1.592**	1.724***	Fin. difficulties x Baltic	1.175***	1.522***	Job ends x Baltic	1.967***	1.707***
	[1.112–2.280]	[1.222–2.434]		[1.111–1.243]	[1.208–1.917]		[1.585–2.442]	[1.502–1.940]
Observations	17,452	17,452	Observations	20,503	20,503	Observations	20,524	20,525
*p*-value of Wald test	0.425	0.034	p-value of Wald test	0.007	0.121	p-value of Wald test	0.081	0.008
*Second group of models*								
	Poor health	Long-term illness		Poor health	Long-term illness		Poor health	Long-term illness
CEE x stress	1.563***	1.966***	CEE x fin. difficulties	1.773***	1.588***	CEE x job ends	1.502***	1.238*
	[1.233–1.980]	[1.473–2.623]		[1.408–2.232]	[1.243–2.029]		[1.368–1.650]	[0.979–1.564]
West x stress	1.526***	1.506***	West x fin. difficulties	1.670***	1.780***	West x job ends	1.270	1.282***
	[1.114–2.088]	[1.343–1.689]		[1.343–2.077]	[1.540–2.057]		[0.931–1.733]	[1.094–1.501]
Observations	35,273	35,276	Observations	43,539	43,541	Observations	43,344	43,344
p-value of Wald test	0.826	0.075	p-value of Wald test	0.731	0.399	p-value of Wald test	0.177	0.707

We control for individual characteristics and country effects. Logit odds ratios are reported. 95% CI displayed in brackets. The Wald test tests the equality of the coefficients of the stress indicators interacted with the country group indicators

*** *p* < 0.01, ** *p* < 0.05, * *p* < 0.1

To analyse to what extent do the associations between transition related shocks and later health vary within the country groups, we estimate a modified version of Eq. (1). Here, we replace *D*_*g*_ with the binary indicators of living in the specific country in the CEE region. Also, we replace *s* with the binary indicator of experiencing any of the analysed three shocks around the transition, with individuals who never had such hardships serving as the comparison group. We analyse the three shocks jointly in the country-specific analysis to ensure that we have a sufficient number of observations of transition related shocks in each country.

In the second group of models, we included the Western countries and analysed a possible interaction between the shocks and the region (CEE versus West) based on the following equation:

2 $$ \Pr \left({\mathrm{h}}_{\mathrm{irc}}=1\right)=\Lambda \left({\mathrm{s}}_{\mathrm{irc}}{\mathrm{D}}_{\mathrm{r}}{\boldsymbol{\upalpha}}_{\mathbf{2}}+{\mathrm{x}}_{\mathrm{irc}}{\boldsymbol{\upbeta}}_{\mathbf{2}}+{\varepsilon}_{\mathrm{c}}\right) $$

where the notation is the same as in Eq. (1), with country-group specific coefficients replaced with region (*r*) specific coefficients. The exponential of **α**_**2**_ (reported in Table 2) shows how the odds of a health problem in 2017 relates to having had hardships during transition in CEE or in the West, with individuals who never had such hardships serving as the comparison group.

In the third group of models (Table 3), we extended the time period and assessed the impact of shocks in CEE between 1984 and 1996, to see whether difficulties around and probably due to the transition are specific or not:

3 $$ \Pr \left({\mathrm{h}}_{\mathrm{ic}}=1\right)=\Lambda \left({\mathrm{s}}_{\mathrm{ic}}{\mathrm{T}}_{\mathrm{ic}}{\boldsymbol{\upalpha}}_{\mathbf{3}}+{\mathrm{x}}_{\mathrm{ic}}{\boldsymbol{\upbeta}}_{\mathbf{3}}+{\omega}_{\mathrm{c}}\right) $$

**Table 3:** Third group of models – Health measures regressed on difficulties occurring between 1987 and 1993 versus 1984–1986 and 1994–1996 in CEE

	Poor health	Long-term illness		Poor health	Long-term illness		Poor health	Long-term illness
transition x stress	1.695***	2.045***	transition x fin. difficulties	1.923***	1.993***	transition x job ends	1.379***	1.441***
	[1.429–2.010]	[1.812–2.306]		[1.515–2.441]	[1.581–2.512]		[1.174–1.620]	[1.189–1.745]
before/after transition x stress	1.562***	1.940***	before/after transition x fin. difficulties	1.780***	1.589***	before/after transition x job ends	1.403***	1.156
	[1.203–2.028]	[1.378–2.731]		[1.428–2.219]	[1.187–2.127]		[1.147–1.715]	[0.804–1.660]
observations	19,174	19,174	observations	21,837	21,837	observations	21,773	21,774

We control for individual characteristics and country effects. Logit odds ratios are reported. 95% CI displayed in brackets

*** *p* < 0.01, ** *p* < 0.05, * *p* < 0.1

where the notation is the same as in Eq. (1), but instead of estimating country-group specific coefficients, we allow the health implications of hardships to vary with the time period when the difficulties occurred, denoted by *T*_*ic*_ (during the transition period versus before or after the transition period).

Finally, we estimated a modified version of Eq. (3), where we allow the health implications of hardships to vary with gender, education and age category, restricting the sample again to CEE and considering shocks occurring between 1987 and 1993 (Table 4).

**Table 4:** Heterogeneity analysis results - Health measures regressed on difficulties occurring between 1987 and 1993 in CEE

	poor health	long-term illness		poor health	long-term illness		poor health	long-term illness
stress	2.206***	2.650***	fin. difficulties	1.910***	1.874***	job ends	1.880***	1.625***
	[1.728–2.817]	[1.742–4.032]		[1.494–2.441]	[1.315–2.669]		[1.466–2.410]	[1.223–2.159]
stress x female	0.701**	0.814	fin. difficulties x female	0.874	0.942	job ends x female	0.736	0.787
	[0.512–0.960]	[0.475–1.394]		[0.679–1.125]	[0.686–1.293]		[0.465–1.166]	[0.500–1.237]
observations	17,452	17,452	observations	20,503	20,503	observations	20,524	20,525
stress	2.707***	3.097***	fin. difficulties	1.629	1.496***	job ends	1.019	1.082
	[1.652–4.434]	[1.573–6.098]		[0.868–3.060]	[1.295–1.729]		[0.881–1.178]	[0.866–1.351]
stress x secondary education	0.655*	0.787	fin. difficulties x secondary edu.	1.090	1.259	job ends x secondary edu.	1.672***	1.383**
	[0.416–1.030]	[0.415–1.493]		[0.599–1.983]	[0.902–1.757]		[1.407–1.988]	[1.070–1.787]
stress x tertiary education	0.645	0.615	fin. difficulties x tertiary edu.	1.157	1.060	job ends x tertiary edu.	1.148	1.017
	[0.321–1.297]	[0.269–1.407]		[0.544–2.461]	[0.649–1.730]		[0.861–1.532]	[0.740–1.398]
observations	17,452	17,452	observations	20,503	20,503	observations	20,524	20,525
stress	1.743***	2.135***	fin. difficulties	1.939***	1.744***	job ends	1.415***	1.342**
	[1.374–2.212]	[1.873–2.433]		[1.185–3.170]	[1.310–2.321]		[1.257–1.593]	[1.030–1.749]
aged< 36 in 1990 x stress	1.092	1.219*	aged< 36 in 1990 x fin. difficulties	0.853	1.066	aged< 36 in 1990 x job ends	1.211**	1.090
	[0.756–1.577]	[0.965–1.540]		[0.517–1.407]	[0.753–1.507]		[1.011–1.451]	[0.694–1.714]
observations	17,452	17,452	observations	20,503	20,503	observations	20,524	20,525

We control for individual characteristics and country effects. Logit odds ratios are reported. 95% CI displayed in brackets

*** *p* < 0.01, ** *p* < 0.05, * *p* < 0.1

## Results

### Shocks around the transition

**Fig. 1:**
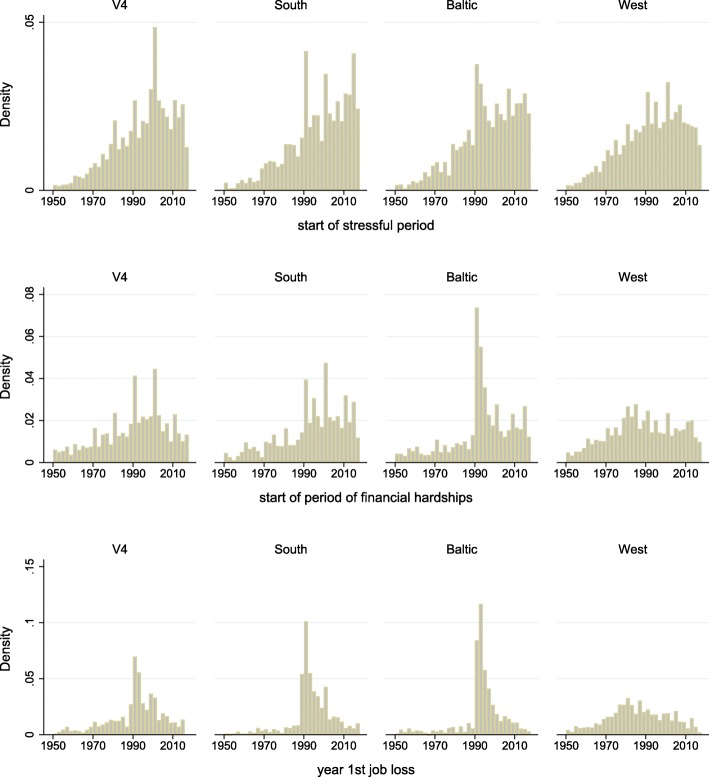
Starting year of stressful periods and financial hardships and end year of 1st job with a 6 months gap or immediate retirement afterwards in CEE and West. Source: SHARE Waves 3 and 7

Looking at the distribution of the start year of periods with difficulties and the year when the first job ends with at least 6 months of gap or retirement afterwards, these have a peak around the transition (1990) in the post-socialist countries, but not in the West (Fig. 1 – in the third panel, for illustration purposes, we plot the year of the first job ending, but in the regression analyses we use a binary indicator if any, thus not only the first job ends around the transition).

The probability that between 1987 and 1993 (i.e. around the transition), someone had a stressful period, a period with financial hardship or an ending of any job with a gap or retirement afterwards is 0.5%point (95% CI: − 0.005; 0.014), 3%points (95% CI: 0.018; 0.038) and 17%points (95% CI: 0.158; 0.185) higher in the post-socialist countries than in the West, respectively, among those who experienced the specific shock ever. Overall 19% (95% CI: 0.183; 0.192) of the participants reported at least one of these three shocks around the transition in CEE compared to 15% (95% CI: 0.151; 0.158) in the West. Among those, who experienced at least one of these shocks ever, 31% (95% CI: 0.308; 0.321) had a shock around the transition in the CEE, as opposed to 24% (95% CI: 0.235; 0.244) in the West. Within the CEE group, this fraction is the highest in the Baltic countries with 33% (95% CI: 0.315; 0.338), followed by the Southern countries with 31% (95% CI: 0.298; 0.321) and the V4 with 27% (95% CI: 0.260; 0.281).

Holding age, gender, education level and the industry of the last job fixed, using West as the comparison country group, and focusing on those who had at least one of the analysed shocks, respondents from the V4, Baltic and Southern states are 0.5%point (95% CI: − 0.018; 0.028), 4.9%points (95% CI: 0.027; 0.071) and 5.3%points (95% CI: 0.029; 0.077) more likely, respectively, to report any of the three analysed shocks occurring between 1987 and 1993.

Also, among those who stopped working in a job between 1987 and 1993 with a gap or retirement afterward, it is more prevalent in the post-socialist countries that the reason for leaving a job was either being laid off or plant/office shut-down (36% in the West, 95% CI: 0.341; 0.375, versus 62% in CEE, 95% CI: 0.603; 0.634).

### Health gap at old age in CEE

Assessing current health of the 50+ population in 2017, we see that self-rated health is on average worse in the CEE countries, but this difference compared to West is more substantial in the Baltic countries than in the Southern and V4 CEE countries (first part of Table 1). Also, most chronic diseases and obesity are more prevalent in CEE, which are known to be influenced by living conditions and lifestyle. However, there are some variations across the CEE country groups, the health status of individuals from the South seems to be on average the most similar to the health of Western people. This is also reflected by the indicators of having any long-term illness and health issues limiting work. On the other hand, based on our data, there is little difference in the prevalence of cancer, chronic lung diseases and weak grip strength between CEE and West. Note, that these statistics are conditional on having survived 20–30 years after the transition. The health differences between the country groups change little if age and gender are controlled for.

### Regression results

The first group of models of current health conditions is estimated on the subsample of CEE countries (upper part of Table 2 and Appendix 1 Table 5), with individuals who never had the analysed hardships serving as the comparison group. The results indicate that a stressful period, financial difficulties and job loss around the transition (between 1987 and 1993) are generally associated with worse later health, accounting for observed individual characteristics (demographics and measures of childhood health) and country heterogeneity. The odds of reporting poor health and having a long-term illness increase with reporting shocks around the transition in all three country groups. The results reported in the Appendix indicate that difficulties around the transition are systematically related to higher likelihood of specific chronic diseases and obesity. There are few exceptions, where a negative association is found between health problems and difficulties around transition, such as heart problem and obesity in the South. However, these negative associations are mostly statistically insignificant.

**Fig. 2:**
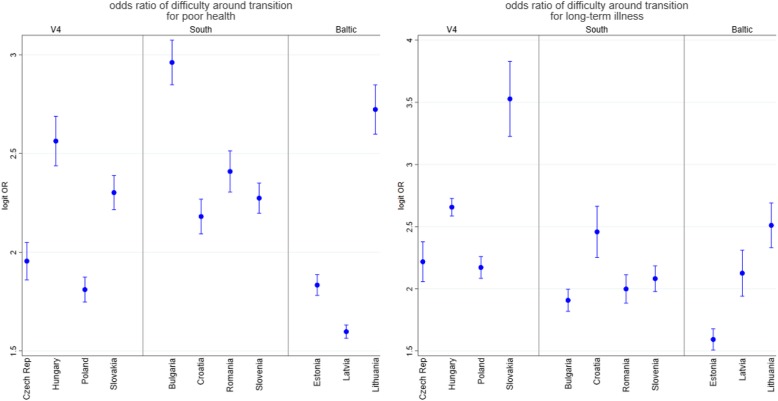
First group of models – Health measures regressed on difficulties occurring between 1987 and 1993 in CEE country groups, with county specific OR. Notes. Logit odds ratios are displayed with 95% CI. The figure displays the OR of reporting any of the analysed three shocks (stress, financial difficulties, job loss with a gap afterwards)

The associations between the difficulties around the transition and later health are broadly similar across the three country groups of CEE, there is no systematic pattern in the differences in the associations. Fig. 2 in the Appendix 2 shows that difficulties around the transition are associated with worse health in each country of CEE, although these associations are heterogeneous even within the three country groups. Overall, the results suggest that the analysed relations are stronger in Slovakia within the V4 countries, and in Lithuania, within the Baltic countries.

In the second group of models (lower part of Table 2 and Appendix 3 Table 6) we did not find clear evidence that the health implications of shocks occurring in CEE and the West would be different. We generally see stronger association between the reported difficulties and later health problems in CEE, but Wald tests of the equality of coefficients indicate that most of these differences are statistically insignificant.

While there is a peak in the timing of difficulties around the transition in CEE, difficulties occurring before or after the transition have similar health implications (Table 3 and Table 7 in the Appendix 4).

We report heterogeneity analysis results in Table 4. The heterogeneities in the health implications of difficulties around transition by gender, education or age group are mostly statistically insignificant. The results suggest that the health implications of difficulties around transition are stronger among men and the younger. The health implications of stress around transition seem to be stronger among the lower educated, whereas the health implications of financial difficulties and job loss around transition are stronger among the more educated.

## Discussion

Using retrospective data, we analysed whether the hardships around the transition contributed to the health gap between post-socialist CEE, and Western Europe. We showed that the era of post-socialist transition was indeed more often associated with the start of stressful periods, financial difficulties and the termination of a job with a gap or immediate retirement afterwards in CEE than in the West. Within the analysed CEE countries, difficulties around the transition were most often reported in the Baltic states, also, respondents from the Baltic states reported on average the poorest health. These results correspond to the macroeconomic evidence that the transition had the most severe economic effect in the Baltic states [39].

We found evidence that stressful periods, financial difficulties and job loss around the period of transition are mostly associated with worse health at older ages in all groups of CEE countries, even after netting out the effect of childhood health and demographic factors, implying that psychosocial stress as an adult around the transition cumulated over the life course into weaker health at older ages. The associations between the three different stressors (stress, financial difficulties and job loss) and later health are comparable, thus they seem to be similarly important to mitigate during the life course.

While the transition was a drastic restructuring of the political-economic systems absent in the West, we found that major difficulties are negatively related to later health both in CEE and the West, indicating that the consequences of hardships due to the transition are not specific, health implications of these difficulties seem to be similar to the implications of other shocks possibly unrelated to the transition, such as an economic crisis. Nevertheless, the transition implied major difficulties for 19% (95% CI: 0.183; 0.192) of the individuals in CEE, whereas in the West only 15% (95% CI: 0.151; 0.158) experienced hardships during the same period. Also, among those who experienced any of the analysed difficulties ever, those difficulties were 7.5%points (95% CI 0.060; 0.091) more likely to occur around the transition in the CEE than in the West. Thus, not the transition-specific nature of the difficulties, but the higher fraction of individuals experiencing them around the transition contributed to the current health disadvantage in CEE.

Heterogeneity analysis revealed that the health implications of difficulties around transition were stronger among males and the younger, although most of the gender and age differences were statistically insignificant. The gender differences are in line with the literature which established that the mortality consequences of transition were stronger among males [6, 7, 40]. Stronger health implications of socio-economic strain among those who were hit by the transition at a younger age point to the risk of accumulating health disadvantage over the life course.

Our study is subject to a set of limitations. The results are conditional on having survived around 20 years after transition, thus we could not estimate the mortality effects. If the mortality rate was higher among those whose health was most affected by the hardships around transition (which is likely to be the case) then the negative health implications of the hardships around transition were even stronger than what our results suggest. Health behaviours could not be analysed due to data limitations. Also, reverse causality is possible from persistent health problems to reporting hardships related to the transition, therefore our results indicate associations rather than causal effects. As an alternative identification, we compared individuals experiencing hardship around the transition with individuals not reporting hardship or facing difficulties only after the transition and these more conservative estimates are in line with the reported results.

## Conclusions

Overall, our results draw the attention to the long-lasting impacts of psychosocial stress and financial hardship during adulthood on later health. Hence, our analysis relates to the literature analysing the effect of the recent financial crisis and austerity on health [41–43].

As the different types of stressors have similar associations with later health, policies that have the potential to alleviate more of these interrelated experiences, such as employment protection or activation policies are worth considering. We suggest including alleviating youth unemployment on the policy agenda given that some of the health implications of difficulties around transition were stronger among the younger. However, further research is needed to formulate suitable policy recommendations, both in terms of policy tools and target groups.

In general, at times of economic recession or wide-spread political and economic restructuring when a large proportion of the population is directly affected by rising insecurity, policy makers should also consider the health implications of their policy responses to mitigate the cumulating health disadvantages over the life-course.

## Data Availability

The datasets analysed during the current study are available in the SHARE repository, the DOIs to SHARE Waves 1, 2, 3, 4, 5, 6 and 7 are the following: 10.6103/SHARE.w1.700, 10.6103/SHARE.w2.700, 10.6103/SHARE.w3.700, 10.6103/SHARE.w4.700, 10.6103/SHARE.w5.700, 10.6103/SHARE.w6.700, 10.6103/SHARE.w7.700), see Börsch-Supan et al. (2013) for methodological details.

## Appendix 1

**Table 5:** First group of models – Further health measures regressed on difficulties occurring between 1987 and 1993 in CEE country groups

	Health limits work	Hyper-tension	Heart problem	Diabetes	Ulcer	Cancer	Lung disease	Obese	Weak grip strength
Stress x V4	1.623***	1.337***	1.346**	1.896***	1.222	1.483	1.699**	1.501***	1.192
	[1.276–2.065]	[1.124–1.591]	[1.055–1.718]	[1.284–2.797]	[0.771–1.938]	[0.920–2.389]	[1.011–2.855]	[1.173–1.919]	[0.878–1.617]
Stress x South	1.916***	1.357***	0.859	0.965	1.965***	1.490**	0.852	1.035	1.101
	[1.812–2.025]	[1.185–1.555]	[0.518–1.422]	[0.792–1.177]	[1.181–3.269]	[1.067–2.080]	[0.486–1.493]	[0.846–1.265]	[0.975–1.243]
Stress x Baltic	1.647***	1.312***	1.195***	1.371	1.500***	1.458	1.307***	1.423***	0.861*
	[1.520–1.784]	[1.177–1.463]	[1.070–1.334]	[0.822–2.287]	[1.304–1.726]	[0.886–2.401]	[1.081–1.582]	[1.198–1.691]	[0.733–1.012]
Observations	14,200	17,393	17,393	17,393	17,393	17,393	17,393	17,087	15,859
Financial difficulties x V4	1.030	1.269**	1.134**	2.059***	1.038	0.779**	2.714**	1.364***	0.852***
	[0.850–1.247]	[1.002–1.608]	[1.001–1.284]	[1.349–3.142]	[0.704–1.532]	[0.643–0.943]	[1.069–6.895]	[1.262–1.474]	[0.764–0.951]
Fin. difficulties x South	1.423	1.431***	0.802	1.115	2.712***	2.197*	1.350	0.866	1.548***
	[0.768–2.639]	[1.286–1.592]	[0.525–1.225]	[0.840–1.479]	[2.332–3.154]	[0.953–5.067]	[0.865–2.108]	[0.678–1.107]	[1.352–1.772]
Fin. difficulties x Baltic	1.370***	1.231***	1.058	1.340	1.321**	0.990	1.143	1.006	1.238**
	[1.301–1.443]	[1.099–1.380]	[0.923–1.213]	[0.937–1.914]	[1.064–1.639]	[0.878–1.115]	[0.797–1.637]	[0.791–1.279]	[1.040–1.472]
Observations	16,631	20,438	20,438	20,438	20,438	20,438	20,438	20,088	18,680
Job ends x V4	1.856***	1.001	1.471***	1.117	1.173	1.540***	1.154**	0.876	1.309**
	[1.582–2.178]	[0.872–1.148]	[1.410–1.535]	[0.899–1.388]	[0.965–1.427]	[1.264–1.875]	[1.012–1.316]	[0.695–1.105]	[1.023–1.675]
Job ends x South	1.497***	0.954	0.777**	1.092	1.505***	1.518	1.668***	0.816**	1.001
	[1.158–1.935]	[0.898–1.013]	[0.626–0.964]	[0.897–1.328]	[1.306–1.733]	[0.847–2.720]	[1.567–1.776]	[0.694–0.959]	[0.881–1.137]
Job ends x Baltic	1.930***	1.111	1.048	1.001	1.533***	1.173**	1.021	1.138	1.122
	[1.795–2.074]	[0.915–1.350]	[0.945–1.162]	[0.820–1.222]	[1.147–2.049]	[1.029–1.337]	[0.843–1.237]	[0.966–1.340]	[0.955–1.318]
Observations	16,598	20,458	20,458	20,458	20,458	20,458	20,458	20,112	18,721

We control for individual characteristics and country effects in CEE. Logit odds ratios are reported. 95% CI displayed in brackets

## Appendix 3

**Table 6:** Second group of models – Further health measures regressed on difficulties occurring between 1987 and 1993 in CEE and West

	health limits work	hyper-tension	heart problem	diabetes	ulcer	cancer	lung disease	obese	weak grip strength
CEE x stress	1.496***	1.283***	1.388**	1.547***	1.237	1.994***	1.280	1.135	1.101
	[1.256–1.783]	[1.123–1.466]	[1.053–1.830]	[1.207–1.982]	[0.822–1.862]	[1.406–2.828]	[0.893–1.833]	[0.814–1.582]	[0.955–1.270]
West x stress	1.629***	1.079	1.075	0.904	1.248*	1.023	1.064	1.022	0.892
	[1.389–1.912]	[0.956–1.219]	[0.892–1.294]	[0.663–1.233]	[0.990–1.573]	[0.898–1.165]	[0.912–1.242]	[0.671–1.556]	[0.760–1.046]
Observations	27,523	35,197	35,197	35,197	35,197	35,197	35,197	34,306	32,167
CEE x financial difficulties	1.163	1.291***	1.411	1.459*	1.871**	1.042	2.096***	1.104	0.937
	[0.921–1.470]	[1.141–1.461]	[0.831–2.394]	[0.947–2.247]	[1.074–3.260]	[0.705–1.541]	[1.259–3.490]	[0.882–1.383]	[0.668–1.313]
West x financial difficulties	1.845***	1.193***	1.024	1.397**	1.485	1.102	1.718***	1.686***	1.220***
	[1.423–2.393]	[1.047–1.360]	[0.673–1.557]	[1.063–1.835]	[0.877–2.515]	[0.625–1.943]	[1.218–2.424]	[1.349–2.107]	[1.068–1.395]
Observations	34,358	43,442	43,442	43,442	43,442	43,442	43,442	42,435	39,954
CEE x job ends	1.531***	1.129	1.255**	1.270***	1.260**	1.152	1.479***	0.911	1.092
	[1.245–1.884]	[0.968–1.316]	[1.053–1.494]	[1.091–1.479]	[1.040–1.526]	[0.789–1.684]	[1.183–1.848]	[0.795–1.043]	[0.849–1.403]
West x job ends	1.302***	0.847	1.042	1.089	1.853***	1.122	1.213*	0.969	1.043
	[1.105–1.535]	[0.634–1.132]	[0.844–1.286]	[0.813–1.460]	[1.352–2.541]	[0.923–1.364]	[0.988–1.489]	[0.823–1.143]	[0.952–1.143]
Observations	34,140	43,246	43,246	43,246	43,246	43,246	43,246	42,273	39,757

We control for individual characteristics and country effects in CEE and West. Logit odds ratios are reported. 95% CI displayed in brackets

*** *p* < 0.01, ** *p* < 0.05, * *p* < 0.1

## Appendix 4

**Table 7:** Third group of models – Further health measures regressed on difficulties occurring between 1987 and 1993 versus 1984–1986 and 1994–1996 in CEE

	health limits work	hyper-tension	heart problem	diabetes	ulcer	cancer	lung disease	obese	weak grip strength
transition x stress	2.084***	0.993	1.349	1.278**	1.696***	1.246	1.356***	1.146	1.173**
	[1.622–2.677]	[0.600–1.643]	[0.849–2.145]	[1.042–1.566]	[1.335–2.153]	[0.436–3.567]	[1.128–1.630]	[0.925–1.420]	[1.017–1.352]
before/after transition x stress	1.565***	1.236***	1.243*	1.408**	1.279	1.733***	1.440**	1.129	1.037
	[1.297–1.889]	[1.060–1.442]	[0.987–1.565]	[1.040–1.907]	[0.860–1.902]	[1.400–2.146]	[1.030–2.014]	[0.793–1.607]	[0.938–1.147]
observations	15,645	19,108	19,108	19,108	19,108	19,108	19,108	18,786	17,507
transition x financial difficulties	2.090***	1.070	1.195	1.484***	1.930***	1.496	1.542*	0.963	0.927
	[1.539–2.837]	[0.763–1.500]	[0.776–1.840]	[1.190–1.851]	[1.382–2.695]	[0.745–3.004]	[0.968–2.458]	[0.644–1.439]	[0.667–1.288]
before/after transition x financial difficulties	1.226	1.211**	1.307	1.362	1.987**	1.007	2.311***	1.087	1.223**
	[0.954–1.576]	[1.014–1.448]	[0.826–2.067]	[0.811–2.287]	[1.051–3.758]	[0.674–1.505]	[1.352–3.950]	[0.856–1.381]	[1.046–1.430]
observations	17,770	21,768	21,768	21,768	21,768	21,768	21,768	21,403	19,951
transition x job ends	1.797***	0.930	1.036	1.393*	1.596***	0.949	1.207	1.053	1.068
	[1.586–2.037]	[0.665–1.300]	[0.609–1.761]	[0.994–1.952]	[1.214–2.097]	[0.657–1.371]	[0.676–2.155]	[0.888–1.250]	[0.821–1.389]
before/after transition x job ends	1.578***	1.001	1.156	1.125*	1.356***	1.094	1.517***	0.891*	1.042
	[1.186–2.100]	[0.942–1.063]	[0.876–1.525]	[0.998–1.269]	[1.112–1.654]	[0.689–1.737]	[1.113–2.069]	[0.786–1.010]	[0.955–1.137]
observations	17,684	21,702	21,702	21,702	21,702	21,702	21,702	21,346	19,913

We control for individual characteristics and country effects. Logit odds ratios are reported. 95% CI displayed in brackets

*** *p* < 0.01, ** *p* < 0.05, * *p* < 0.1

## Abbreviations

CEE: Central and Eastern Europe
CI: confidence interval
ISCED-97: International Standard Classification of Education of 1997
SHARE: Survey of Health, Ageing and Retirement in Europe
V4: Visegrad countries (Czech Republic, Hungary, Poland, Slovak Republic)

## Footnotes

1 This paper uses data from SHARE Waves 1, 2, 3, 4, 5, 6 and 7 (DOIs: 10.6103/SHARE.w1.700, 10.6103/SHARE.w2.700, 10.6103/SHARE.w3.700, 10.6103/SHARE.w4.700, 10.6103/SHARE.w5.700, 10.6103/SHARE.w6.700, 10.6103/SHARE.w7.700), see Börsch-Supan et al. (2013) for methodological details. The SHARE data collection has been primarily funded by the European Commission through FP5 (QLK6-CT-2001-00360), FP6 (SHARE-I3: RII-CT-2006-062193, COMPARE: CIT5-CT-2005-028857, SHARELIFE: CIT4-CT-2006-028812) and FP7 (SHARE-PREP: N°211,909, SHARE-LEAP: N°227,822, SHARE M4: N°261,982). Additional funding from the German Ministry of Education and Research, the Max Planck Society for the Advancement of Science, the U.S. National Institute on Aging (U01_AG09740-13S2, P01_AG005842, P01_AG08291, P30_AG12815, R21_AG025169, Y1-AG-4553-01, IAG_BSR06–11, OGHA_04–064, HHSN271201300071C) and from various national funding sources is gratefully acknowledged (see www.share-project.org).

## References

[1] OECD/EU: Health at a Glance: Europe 2018: State of health in the EU cycle, OECD publishing, Paris; 2018 doi: 10.1787/health_glance_eur-2018-en.

[2] Laaksonen M, McAlister AL, Laatikainen T, Drygas W, Morava E, Nussel E (2001). Do health behaviour and psychosocial risk factors explain the European east-west gap in health status?. Eur J Pub Health.

[3] Steptoe A, Wardle J (2001). Health behaviour, risk awareness and emotional well-being in students from Eastern Europe and Western Europe. Soc Sci Med.

[4] Kornai J (2006). The great transformation of central eastern Europe. Econ Transit.

[5] Scheiring G, Irdam D, King LP (2019). Cross-country evidence on the social determinants of the post-socialist mortality crisis in Europe: a review and performance-based hierarchy of variables. Sociol Health Illn.

[6] Cornia GA. The mortality crisis in transition economies. IZA World Labor. 2016;298:1-10.

[7] Azarova A, Irdam D, Gugushvili A, Fazekas M, Scheiring G, Horvat P (2017). The effect of rapid privatisation on mortality in mono-industrial towns in post-soviet Russia: a retrospective cohort study. Lancet Public Health.

[8] Kornai J (1994). Transformational recession: the main causes. J Comp Econ.

[9] Sachs JD (1996). The transition at mid decade. Am Econ Rev.

[10] Hodgson GM (2006). Institutions, recessions and recovery in the transitional economies. J Econ Issues.

[11] Balcerowicz L. Common fallacies in the debate on the transition to a market economy. Econ Policy. 1994;9(19):18–50.

[12] Popov V (2000). Shock therapy versus gradualism: the end of the debate (explaining the magnitude of transformational recession). Comp Econ Stud.

[13] Godoyv S, Stiglitz JE (2007). Growth, initial conditions, law and speed of privatization in transition countries: 11 years later. transition and beyond.

[14] Bohle D, Greskovits B (2012). Capitalist diversity on Europe’s periphery.

[15] Zatonski W (2007). The east-west health gap in Europe—what are the causes?. Eur J Pub Health.

[16] King L, Hamm P, Stuckler D (2009). Rapid large-scale privatization and death rates in ex-communist countries: an analysis of stress-related and health system mechanisms. Int J Health Serv.

[17] Elder GH, Johnson MK, Crosnoe R (2003). The emergence and development of life course theory. handbook of the life course.

[18] Mayer KU (2009). New directions in life course research. Annu Rev Sociol.

[19] Dannefer D (2003). Cumulative advantage/disadvantage and the life course: cross-fertilizing age and social science theory. J Gerontol B Psychol Sci Soc Sci.

[20] Ferraro KF, Shippee TP, Schafer MH. Cumulative inequality theory for research on aging and the life course. In Bengston VL, Gans D, Pulney NM, Silverstein M (Eds.). New York: Handbook of theories of aging; 2009. (pp. 413-433). Springer Publishing Co.

[21] Corna LM (2013). A life course perspective on socioeconomic inequalities in health: a critical review of conceptual frameworks. Adv Life Course Res.

[22] Pearlin LI, Schieman S, Fazio EM, Meersman SC (2005). Stress, health, and the life course: some conceptual perspectives. J Health Soc Behav.

[23] Lynch SM (2008). Race, socioeconomic status, and health in life-course perspective: introduction to the special issue. Res Aging.

[24] Lynch JW, Kaplan GA, Shema SJ (1997). Cumulative impact of sustained economic hardship on physical, cognitive, psychological, and social functioning. N Engl J Med.

[25] Pearlin LI, Menaghan EG, Lieberman MA, Mullan JT (1981). The stress process. J Health Soc Behav.

[26] Lazareva O (2020). The effect of labor market shocks on health: the case of the Russian transition. Econ Hum Biol.

[27] Dingsdale A. Redefining ‘Eastern Europe’: a new regional geography of post-socialist Europe? Geography. 1999;84(3):204–21.

[28] Börsch-Supan A, Brandt M, Hunkler C, Kneip T, Korbmacher J, Malter F (2013). Data resource profile: the survey of health, ageing and retirement in Europe (SHARE). Int J Epidemiol.

[29] Börsch-Supan A. Survey of Health, Ageing and Retirement in Europe (SHARE) Wave 3 – SHARELIFE. Release version: 7.0.0. SHARE-ERIC. 2019. Data set. doi: 10.6103/SHARE.w3.700.

[30] Börsch-Supan A. Survey of Health, Ageing and Retirement in Europe (SHARE) Wave 7. Release version: 7.0.0. SHARE-ERIC. 2019. Data set. doi: 10.6103/SHARE.w7.700.

[31] Idler EL, Benyamini Y. Self-rated health and mortality: a review of twenty-seven community studies. J Health Soc Behav. 1997;38(1):21–37. 9097506

[32] Manor O, Matthews S, Power C (2000). Dichotomous or categorical response? Analysing self-rated health and lifetime social class. Int J Epidemiol.

[33] Subramanian SV, Huijts T, Avendano M (2010). Self-reported health assessments in the 2002 world health survey: how do they correlate with education?. Bull World Health Organ.

[34] Crimmins EM, Kim JK, Solé-Auró A (2011). Gender differences in health: results from SHARE, ELSA and HRS. Eur J Pub Health.

[35] Boerma T, Hosseinpoor AR, Verdes E, Chatterji S (2016). A global assessment of the gender gap in self-reported health with survey data from 59 countries. BMC Public Health.

[36] Rantanen T, Guralnik JM, Foley D, Masaki K, Leveille S, Curb J (1999). Midlife hand grip strength as a predictor of old age disability. JAMA..

[37] Cameron AC, Trivedi PK (2009). Microeconometrics using Stata.

[38] Malter F, Börsch-Supan A (2015). Share wave 5: innovations & methodology. Munich Center for the Economics of Ageing (MEA) at the Max Planck Institute for Social Law and Social Policy.

[39] Milanovic B (1998). Income, inequality, and poverty during the transition from planned to market economy.

[40] Brainerd E (2001). Economic reform and mortality in the former Soviet Union: a study of the suicide epidemic in the 1990s. Eur Econ Rev.

[41] Gili M, Roca M, Basu S, McKee M, Stuckler D (2013). The mental health risks of economic crisis in Spain: evidence from primary care centres, 2006 and 2010. Eur J Pub Health.

[42] Karanikolos M, Mladovsky P, Cylus J, Thomson S, Basu S, Stuckler D (2013). Financial crisis, austerity, and health in Europe. Lancet..

[43] Stuckler D, Reeves A, Loopstra R, Karanikolos M, McKee M (2017). Austerity and health: the impact in the UK and Europe. Eur J Public Health.

